# Chlorogenic Acid Stimulates Glucose Transport in Skeletal Muscle via AMPK Activation: A Contributor to the Beneficial Effects of Coffee on Diabetes

**DOI:** 10.1371/journal.pone.0032718

**Published:** 2012-03-07

**Authors:** Khang Wei Ong, Annie Hsu, Benny Kwong Huat Tan

**Affiliations:** Department of Pharmacology, Yong Loo Lin School of Medicine, National University of Singapore, Singapore, Singapore; University of Las Palmas de Gran Canaria, Spain

## Abstract

Chlorogenic acid (CGA) has been shown to delay intestinal glucose absorption and inhibit gluconeogenesis. Our aim was to investigate the role of CGA in the regulation of glucose transport in skeletal muscle isolated from db/db mice and L6 skeletal muscle cells. Oral glucose tolerance test was performed on db/db mice treated with CGA and soleus muscle was isolated for 2-deoxyglucose transport study. 2DG transport was also examined in L6 myotubes with or without inhibitors such as wortmannin or compound c. AMPK was knocked down with AMPKα1/2 siRNA to study its effect on CGA-stimulated glucose transport. GLUT 4 translocation, phosphorylation of AMPK and Akt, AMPK activity, and association of IRS-1 and PI3K were investigated in the presence of CGA. In db/db mice, a significant decrease in fasting blood sugar was observed 10 minutes after the intraperitoneal administration of 250 mg/kg CGA and the effect persisted for another 30 minutes after the glucose challenge. Besides, CGA stimulated and enhanced both basal and insulin-mediated 2DG transports in soleus muscle. In L6 myotubes, CGA caused a dose- and time-dependent increase in glucose transport. Compound c and AMPKα1/2 siRNA abrogated the CGA-stimulated glucose transport. Consistent with these results, CGA was found to phosphorylate AMPK and ACC, consistent with the result of increased AMPK activities. CGA did not appear to enhance association of IRS-1 with p85. However, we observed activation of Akt by CGA. These parallel activations in turn increased translocation of GLUT 4 to plasma membrane. At 2 mmol/l, CGA did not cause any significant changes in viability or proliferation of L6 myotubes. Our data demonstrated for the first time that CGA stimulates glucose transport in skeletal muscle via the activation of AMPK. It appears that CGA may contribute to the beneficial effects of coffee on Type 2 diabetes mellitus.

## Introduction

Regular consumption of coffee has been associated with a lower risk of Type 2 diabetes mellitus and it has been replicated across sexes, geographical locations and obesity levels [1], [2], [3], [4], [5], [6]. However, Battram et al. (2006) showed that the area under the curve (AUC) of glucose was significantly lowered during an oral glucose tolerance test (OGTT) following consumption of decaffeinated coffee compared with caffeinated coffee and a placebo [7]. In addition, with both OGTT [7], [8], [9], [10] and euglycemic-hyperinsulinemic clamp [9], [11], [12], [13] techniques, caffeine had been shown to impair insulin sensitivity. These findings suggest that the beneficial effects of coffee consumption on diabetes may be caused by compounds other than caffeine.

Besides caffeine, coffee contains numerous compounds like phenols, diterpenes, trigonelline and minerals such as potassium and magnesium. Among them, chlorogenic acid [14], [15], [16], [17], trigonelline [14], quinides [18] and magnesium [19] have been shown to affect glucose metabolism. Chlorogenic acid (CGA), one of the phenols in coffee, is the second major component in coffee after caffeine. It is an ester formed from cinnamic acids and quinic acid and is also known as 5-O-caffeoylquinic acid (5-CQA) (IUPAC numbering) or 3-CQA (pre-IUPAC numbering) [20]. It has been shown to delay glucose absorption in the intestine through inhibition of glucose-6-phosphate translocase 1 and reduction of the sodium gradient-driven apical glucose transport [16]. Besides, CGA and its derivatives decreased hepatic glucose output through inhibition of the activity of glucose-6-phosphatase (G-6-Pase) [15], [17], [21].

Van Dijk et al. (2009) showed that CGA ingestion significantly reduced early fasting glucose and insulin responses in overweight men during an OGTT [14]. However, improvement in fasting glucose and insulin cannot be explained by the delay in intestinal glucose absorption. At the same year, there was another study showing that CGA enhanced glucose uptake in skeletal muscle cells [22]. Hence, we propose that CGA stimulates peripheral glucose disposal and thus improving fasting glucose profile. In the present study, we study the effects of CGA on oral glucose tolerance test performed in db/db mice. We also investigate the effect of CGA on 2-deoxyglucose (2-DG) transport by skeletal muscle (*in vitro* and *ex vivo*) and its possible mechanism(s) of this effect. We investigated the effect of inhibitors such as wortmannin and compound c on 2-DG uptake stimulated by CGA. This is followed by the study on the expressions of insulin signaling and AMP-activated protein kinase (AMPK)-dependent pathway proteins involved in the translocation of glucose transporter 4 (GLUT 4). In addition, AMPK was knocked down to investigate its role in CGA-stimulated 2-DG uptake. We also examined the effect of CGA on cell viability and proliferation.

## Materials and Methods

### Materials

CGA, DMEM, Krebs-Ringer bicarbonate buffer (KRBB), antibiotic/antimycotic, insulin, wortmannin, cytochalasin B, MTT, AMP, PI/RNase were obtained from Sigma (St. Louis, MO, USA). Rat L6 skeletal muscle myoblasts were obtained from ATCC (Manassas, VA, USA). FBS was from Hyclone (Cramlington, UK). DMSO was purchased from MP Biomedicals (Illkirch, France). Glucose oxidase kits were obtained from Thermo Scientific (Waltham, MA, USA). Compound c and NP 40 were obtained from Merck (Darmstadt, Germany). 2-Deoxy-[^3^H]D-glucose and [γ-^32^P]-ATP were purchased from PerkinElmer (Waltham, MA, USA). Protease inhibitor cocktail was purchased from Abcam (Cambridge, UK). AMPK α1/2 and an unrelated siRNA (control siRNA-A) were purchased from Santa Cruz (Santa Cruz, CA). Antibodies like anti-IRS-1, anti-PI3-kinase p85α, anti-GLUT 4, anti-GLUT 1, anti-CAMKKβ, anti-phospho-AMPK α1/2, anti-phospho-Akt1 were obtained from Santa Cruz (Santa Cruz, CA) too. anti-GAPDH and anti-phospho-ACC were from Cell Signaling Technology (Danvers, MA, USA) and Milipore (Billerica, MA, USA) respectively. Oligofectamine and OPTI-MEM were purchased from Invitrogen (Carlsbad, CA, USA). Bradford protein estimation kit was from Bio-Rad (Hercules, CA, USA). G-Sepharose beads and ECL detection kit were obtained from GE Healthcare (Piscataway, NJ, USA). SAMS peptide was purchased from Tocris Bioscience (Minneapolis, MN, USA).

### Experimental animals

Thirty male *db/db* mice homozygous for diabetes spontaneous mutation (Lepr^db^) were obtained from The Jackson Laboratory (Sacramento, CA, USA). Ten C57BL/6 mice were purchased from Centre for Animal Resources (CARE), National University of Singapore. They were allowed to acclimatize to conditions in the Animal Holding Unit (AHU), NUS. They were housed throughout the experiment on a 12-hour light/dark cycle. Water and feeds were available to the animals ad libitum.

### Ethics statement

The Principles of Laboratory Animal Care (NIH, 1985) were followed throughout the duration of experiment. The experimental protocol for animal study was approved by NUS Institutional Animal Care and Use Committee (IACUC) (Protocol No: 085/07(A3)10).

### Oral glucose tolerance test

Twenty *db/db* mice were randomly assigned into four groups (n = 4) and four C57BL/6 mice were assigned as lean control group. They were fasted for six hours before the test. Blood samples were collected from the tail vein for fasting glucose measurement using glucose oxidase method before treatments (vehicle, ip 250 mg/kg CGA, oral 250 mg/kg metformin). Ten minutes after the treatments, blood samples were collected again followed by oral gavaging of 2 g/kg glucose. Blood samples were collected 15, 30, 60 and 120 minutes after the glucose challenge.

### 2DG transport in soleus muscle

Soleus muscle was isolated from db/db mice as described previously [23]. It was then incubated with treatments (vehicle, metformin or CGA) in KRBB for 30 minutes at 37°C. Treated muscle strips were subsequently incubated with 0.5 ml KRBB containing 1 µCi/ml 2-Deoxy-[^3^H]D-glucose for 30 minutes at 37°C. Reaction was terminated by immediately blotting the tissues and dissolving them in 0.5 N NaOH for an hour followed by neutralization with equal amount of 0.5 N HCL. After centrifugation, supernatant was collected for quatitation of 2DG taken up by the tissue using liquid scintillation counter (Beckman Coulter LS6500 Multi-Purpose Scintillation Counter, Fullerton, CA, USA). Non-specific uptake was measured in the presence of 10 µmol/l cytochalasin B and subtracted from the total uptake. 2-DG uptake was expressed as a percentage of the basal uptake of cells incubated with KRBB buffer only.

### Cell culture and differentiation of L6 skeletal muscle

The culture was maintained in DMEM containing 10% FBS and 1% antibiotic/antimycotic in a humidified atmosphere of 5% CO_2_ at 37°C. Differentiation of myoblasts into myotubes was carried out as described by Klip et al. [24]. L6 myoblast was seeded in 10% FBS-DMEM until it reached 80–90% confluence; the FBS content was reduced to 2% for a further 5–7 days to induce myotube formation. The degree of differentiation was determined as the percentage of nuclei present in the multinucleated myotubes under a phase-contrast microscope. Before all experimental manipulations, L6 myotubes were deprived of serum for 4 hours to render the cells quiescent.

### 2-Deoxy-[^3^H]D-glucose (2-DG) transport in L6 skeletal muscle

The cells were grown and differentiated in 96-well plates. After the indicated periods of incubation with different treatments, the cells were rinsed with KRPH (HEPES-buffered Krebs-Ringer phosphate) buffer, consisting of 118 mmol/l NaCl, 5 mmol/l KCl, 1.3 mmol/l CaCl_2_, 1.2 mmol/l MgSO_4_, 1.2 mmol/l KH_2_PO_4_ and 30 mmol/l HEPES (pH 7.4). CGA was prepared in 5% DMSO and diluted with appropriate amount of DMEM to obtain different concentrations. 5% DMSO was used for all drug preparations. For the study with CGA+insulin, myotubes were treated with 2 mmol/l CGA for 24 hours and stimulated with 100 nmol/l insulin for 30 mins before 2DG uptake measurement. For the study involving inhibitors, myotubes were pre-incubated with 100 nmol/l wortmannin or 10 µmol/l compound c for 30 mins before adding CGA. Washed cells were incubated with 10 µmol/l 2-Deoxy-[^3^H]D-glucose (1 µCi/ml) in KRPH buffer for 30 mins at 37°C. The 2-DG uptake was terminated immediately by aspirating the radioactive incubation solution and washing three times by ice-cold phosphate-buffered saline (PBS). The cells were then lysed with 0.5 N NaOH, followed by 0.5 N HCL for neutralization. The quantity of 2-DG taken up by the cells was measured with liquid scintillation counter. Non-specific uptake was measured in the presence of 10 µmol/l cytochalasin B and subtracted from the total uptake. 2-DG uptake was expressed as a percentage of the basal uptake of cells incubated with KRPH buffer only.

### Myotube subcellular fractionation

The subcellular fractionation of myotubes was done as previously described [25]. Treated cells were scraped gently from 10 cm dishes and centrifuged at 700 g, 4°C for 10 mins. The pellet was then resuspended in sucrose buffer, consisting of 250 mmol/l sucrose, 5 mmol/l sodium azide, 2 mmol/l EGTA, 20 mmlol/l HEPES (pH 7.4) and protease inhibitor cocktail, and homogenized with 20 strokes using a Dounce homogenizer. The homogenate was centrifuged at 760 g, 4°C for 5 mins to remove nuclei and cell debris. The supernatant was collected as total cell lysate. For plasma membrane isolation, the supernatant was removed and centrifuged at 31,000 g, 4°C for 60 mins to pellet the crude plasma membrane (PM). PM fraction was resuspended in sucrose buffer and stored at −80°C.

### siRNA transfection of myotubes

Transfection of siRNA into myotubes was done as described previously with a minute modification [26]. Cells were seeded and grown in 6-well plates as described earlier (see section on cell culture and differentiation). After cells were treated with 2% FBS for 3 days, siRNAs were transfected with oligofectamine, acoording to the instructions of the manufacturer. Briefly, the complex mixture in serum- and antibiotic-free OPTI-MEM was layered onto the cells and incubated for 4 hours at 37°C. The serum content was then brought up to 2% by adding half volume of antibiotic-free DMEM supplemented with 6% FBS and incubated for an additional 20 hours. Another volume of 2%-FBS DMEM with antibiotic was then added and incubated for further 24 hours to allow the siRNA to remain on the cells for a total of 48 hours.

### Immunoprecipitation and detection of association between IRS-1 and p85 subunit of PI3K

Treated cells were scraped gently from 6-well plate and pelleted with ice-cold PBS at 3,000 rpm, 4°C for 5 minutes. Cell pellet was then lysed in lysis buffer (50 mmol/l Tris [pH 8], 170 mmol/l NaCl, 1 mmol/l DTT, 0.5% NP40 and protease inhibitor cocktail for 30 minutes at 4°C. Cell lysate was then centrifuged at 13,000 rpm for 10 minutes to remove cell debris. 2 µl of the cell lysate was used for Bradford protein estimation. 1 mg of total cellular protein was immunoprecipitated with 1 µg of anti-IRS-1 antibody coupled to 40 µl of 100 mg/ml protein G-Sepharose beads. Immunoprecipitated proteins, together with the beads, were separated with SDS-PAGE and blotted as mentioned in the western blot analysis below. Blotted proteins were probed with anti-PI3-kinase p85α antibody.

### Western blot analysis

Treated cells were scraped from 6-well plates and pelleted with ice-cold PBS at 3,000 rpm, 4°C for 5 minutes. Cell pellets were then lysed in lysis buffer containing 250 mmol/l sucrose, 150 mmol/l NaCl, 50 mmol/l HEPES (pH 7.4) 10 mmol/l sodium fluoride, 1 mmol/l sodium pyrophosphate, 1 mmol/l sodium orthovanadate, 1 mmol/l DTT, 0.5 mmol/l EDTA, 1% Triton-X 100 and proteases inhibitors cocktail. Cell lysates were separated via SDS-PAGE and the separated proteins were blotted onto nitrocellulose membrane. Membranes were probed with anti-GAPDH, anti-GLUT 4, anti-GLUT 1, anti-phospho-AMPK α1/2, anti-phospho-Akt1 and anti-phospho-ACC. They were then developed using the ECL detection kit.

### Cellular viability and proliferation analysis

Myotube viability and proliferation were assayed with MTT and propidium iodode (PI) staining. For the MTT assay, cells were treated with different concentrations of CGA for 24 hours and then incubated with 5 mg/ml MTT for a fruther 4 hours at 37°C. Medium was then removed and 100 µl of DMSO was added to dissolve the formazan crystal formed. Absorbance was measured at a wavelength of 500 nm with a microplate reader (Tecan Infinite m200, Mannedorf, Switzerland). Similarly, for the PI staining, cells were trypsinized and washed with PBS after incubation with different concentrations of CGA for 24 hours. The cells were then fixed in 70% ethanol at 4°C for at least 2 hours. After fixation, the cells were stained with PI/RNase in PBS supplemented with 1% FBS at room temperature for 20 mins before proceeding to flow cytometry analysis, using cyAn ADP Flow Cytometer (Beckman Coulter, Brea, CA).

### AMPK activity assay

Myotubes were treated with CGA of different concentrations for indicated periods of time. Treated cells were scraped and pelleted with ice-cold PBS at 3,000 rpm, 4°C for 5 minutes. Cell pellets were then lysed in lysis buffer containing 250 mmol/l sucrose, 150 mmol/l NaCl, 50 mmol/l HEPES (pH 7.4) 10 mmol/l sodium fluoride, 1 mmol/l sodium pyrophosphate, 1 mmol/l sodium orthovanadate, 1 mmol/l DTT, 0.5 mmol/l EDTA, 1% Triton-X 100 and proteases inhibitors cocktail. 2 µl of the cell lysate was used for Bradford protein estimation. 1 mg of total cellular protein was immunoprecipitated with 1 µg of anti-AMPK α1/2 antibody coupled to 40 µl of 100 mg/ml protein G-Sepharose beads. Kinase reaction was carried out on washed immunoprecipitate in 40 mmol/l HEPES (pH 7.0), 0.2 mmol/l AMP, 80 mmol/l NaCl, 0.8 mmol/l DTT, 5 mmol/l MgCl_2_, 0.2 mmol/l ATP (2 mCi [γ-ATP]) and 0.1 mmol/l SAMS peptide for 20 minutes at 37°C. Reaction mixture was then spotted on P81 Whatman filter paper and washed extensively using phosphoric acid and acetone. Radioactivity on the filter paper was measured using liquid scintillation. Kinase activity was expressed as incorporated ATP/mg protein/minute.

### Statistical analysis

Experiments were repeated three times, each time in triplicate. Values are expressed as mean ± SE. One-way ANOVA followed by Tukey's *t* test was used to determine significant differences between groups. *P* values<0.05 were interpreted as statistically significant.

## Results

### CGA decreased fasting blood glucose in db/db mice

**Figure 1:**
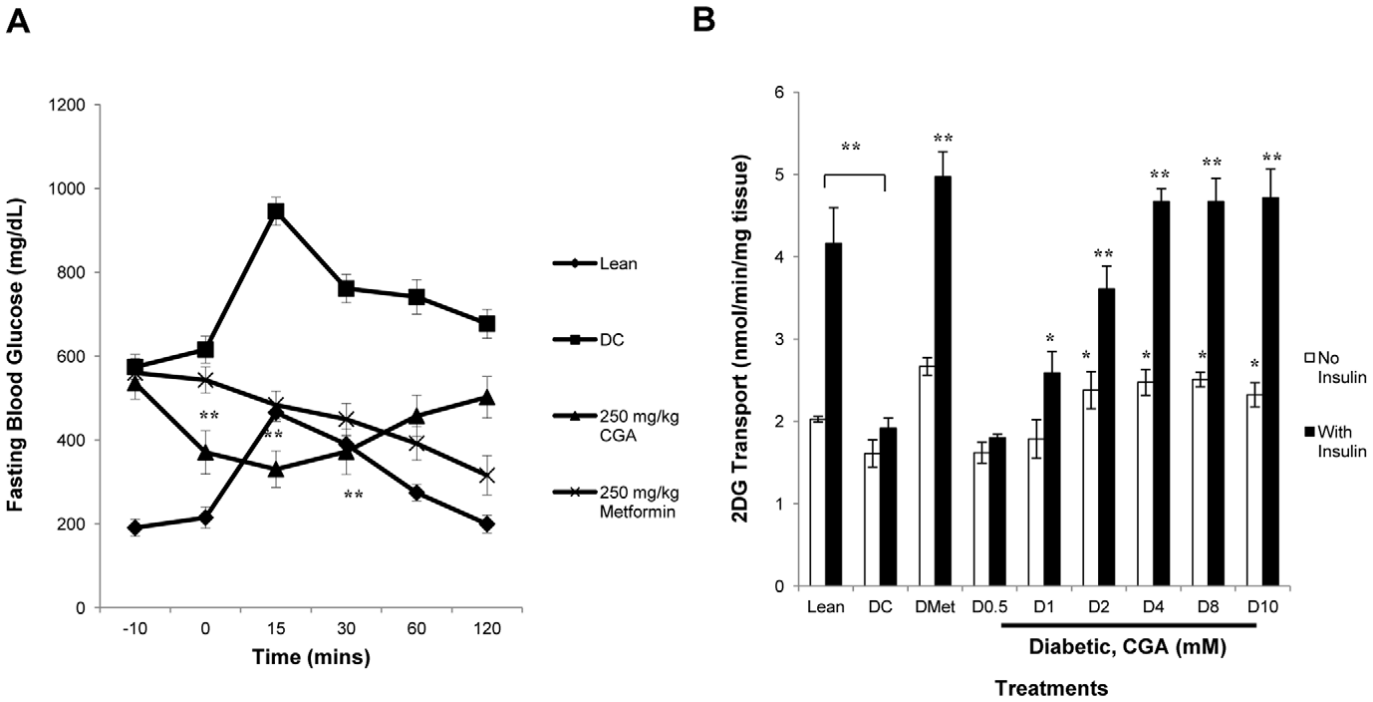
CGA lowered fasting blood glucose in db/db mice and stimulated glucose transport into soleus muscle. A: Oral glucose tolerance test was performed on db/db mice (n = 4) treated with different treatments. 2 g/kg glucose was loaded at 0 minute. Blood samples were collected at −10, 0, 15, 30 60 and 120 minutes for glucose measurement. Results are the mean ± SD of four mice. B: Soleus muscle was isolated from db/db mice and treated with CGA or/and 100 nmol/l insulin or 2 mmol/l metformin for 30 minutes. 2-deoxyglucose uptake was measure over a 30-minute period, using liquid scintillation counter. Results are the mean ± SE of three independent experiments. *P<0.05, **P<0.01 compared with controls. DC = Diabetic Control.

In an oral glucose tolerance test, 250 mg/kg CGA was found to significantly lower fasting blood glucose for 31±9% compared to diabetic control 10 minutes after the intraperitoneal injection (Fig. 1A). This glucose-lowering effect persisted up to 30 minutes and gradually diminished when approaching 60 minutes after the glucose loading. Optimum effect with a significant decrease of 38±9% was observed at 15 minutes after the glucose loading.

### CGA stimulated glucose transport in soleus muscle isolated from db/db mice

Soleus muscle isolated from db/db mice showed a significant increase in glucose transport after treated with CGA (Fig. 1B). 2 mmol/l of CGA increased the glucose transport of myotubes by 48±13% and the stimulation continued to increase and achieve stability at higher concentrations. No significant difference in glucose transport was observed between lean mice and diabetic mice. However, a significant decrease of 54±3% in insulin-stimulated glucose transport was shown in diabetic mice compared to lean mice. CGA treatment further enhanced the insulin-mediated glucose transport for up to 145±13% at the concentrations of 4,8 and 10 mmol/l compared to diabetic control.

### Dose- and time-dependent stimulation of glucose transport by CGA in L6 skeletal muscle cells

**Figure 2:**
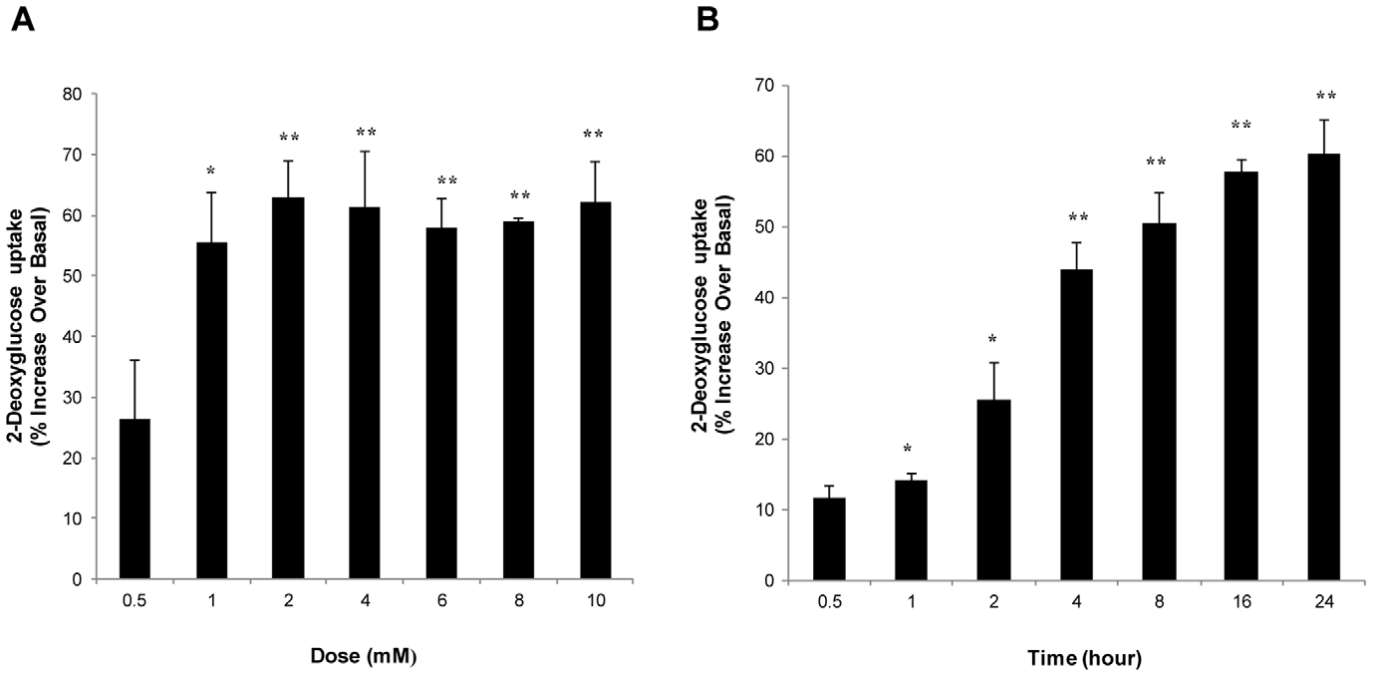
Dose- and time-dependent stimulation of glucose transport in L6 myotubes by CGA. A: L6 myotubes were incubated with incremental concentrations of CGA for 24 hours. B: L6 myotubes were incubated with 2 mmol/l CGA at different incubation periods up to 24 hours. 2-deoxyglucose uptake was measure over a 30-minute period, using liquid scintillation counter. Readings are expressed as percentage increase over basal uptake of cells incubated with vehicle. Results are the mean ± SE of three independent experiments. *P<0.05, **P<0.01 compared with vehicle-treated control.

Treatment of myotubes with increasing concentrations of CGA for 24 hours led to a significant dose-dependent increase in glucose transport, which was first observed at 1 mmol/l (55±8%). The highest increase was observed at 2 mmol/l (63±6%) and the stimulation was maintained up to the highest concentration tested, which was 10 mmol/l (Fig. 2A). Besides, a time-dependent stimulation of glucose transport was also observed when myotubes were incubated with 2 mmol/l CGA at different incubation periods. Significant increase was first observed after 1-hour incubation (14±1%), and continued to increase to 60±5% after 24 hours of incubation (Fig. 2B). In all subsequent experiments, the effects of CGA were determined at a concentration of 2 mmol/l and incubation period of 24 hours.

### Compound c diminished glucose transport stimulated by CGA

**Figure 3:**
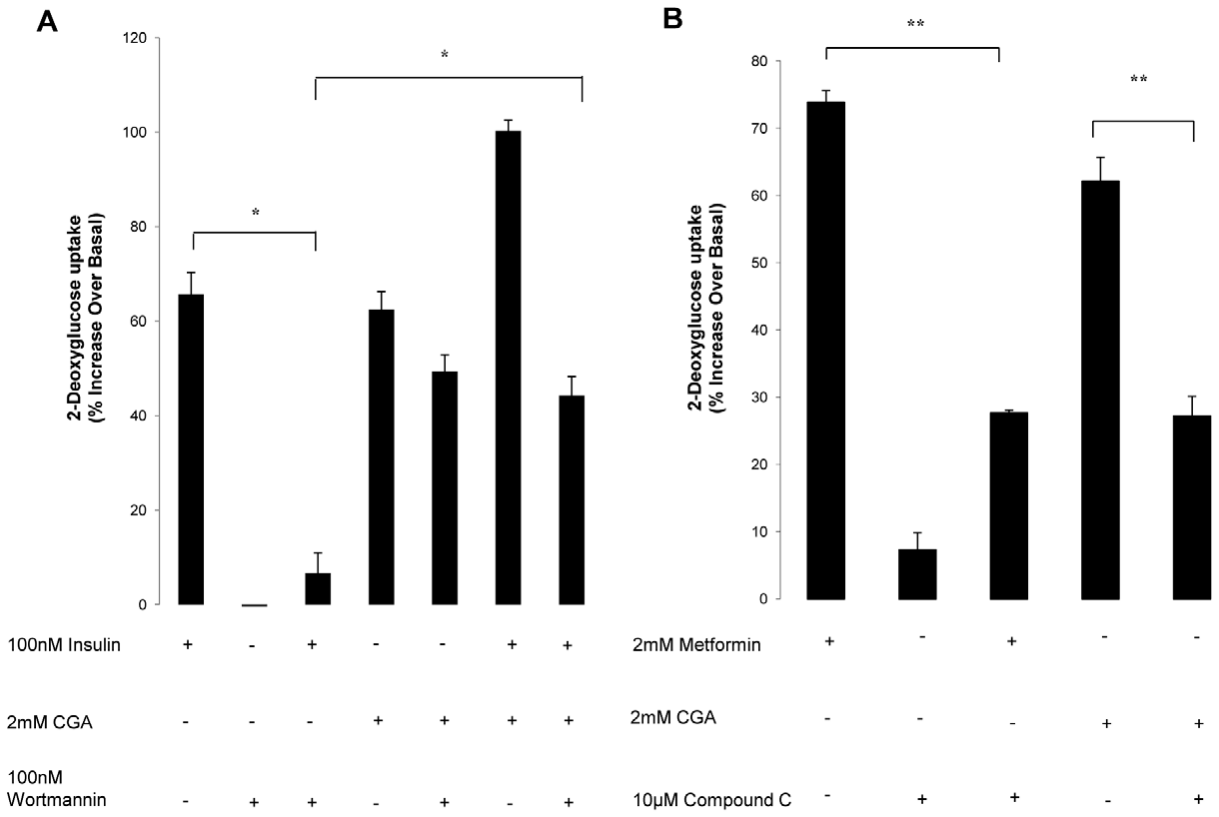
Effects of compound c on CGA-stimulated glucose transport. L6 myotubes were incubated with 2 mmol/l CGA for 24 hours. A: Myotubes were preincubated with 100 nmol/l wormannin for 30 minutes before incubated with CGA or insulin. Myotubes were then incubated with 100 nmol/l insulin 30 minutes before 2-deoyglucose uptake measurement. B: Myotubes were preincubated with 10 µmol/l compound c for 30 minutes before incubated with CGA or metfformin. Myotubes were then incubated with 2 mmol/l metformin 2 hours before 2-deoyglucose uptake measurement. 2-deoxyglucose uptake was measured over a 30-minute period using liquid scintillation counter. Readings are expressed as percentage increase over basal uptake of cells incubated with vehicle. Results are the mean ± SE of three independent experiments. *P<0.05, **P<0.01 compared with controls.

To explain the mechanism underlying the glucose transport stimulated by CGA, we examined the effect of several molecules which are capable of mediating the stimulation of glucose transport. Wortmannin is a well-known selective inhibitor for phosphatidylinositol 3-kinase (PI3K), a key regulator in insulin signaling [27]. Pretreatment of myotubes with 100 nmol/l wortmannin for 30 minutes abolished the stimulatory effect of insulin on glucose transport (Fig. 3A). However, no effect was observed with the wortmannin pretreatment on CGA-stimulated glucose transport. CGA further increased insulin-stimulated glucose transport its glucose transport up to 100±2%, suggesting that CGA may not work on the insulin-dependent pathway. Moreover, pretreatment of CGA and insulin-stimulated transport with wortmannin only partially inhibited the stimulation but not to the extent of that obtained in insulin-only-stimulated transport. Hence, to investigate whether CGA acts through the insulin-independent pathway, we examined the effect of compound c, a selective inhibitor of AMP-activated protein kinase (AMPK) [28], [29], on the CGA-stimulated glucose transport. Pretreatment with 10 µmol/l compound c for 30 minutes significantly abated the glucose transport stimulated by both metformin and CGA (Fig. 3B).

### AMPK is necessary for the glucose transport stimulation by CGA

**Figure 4:**
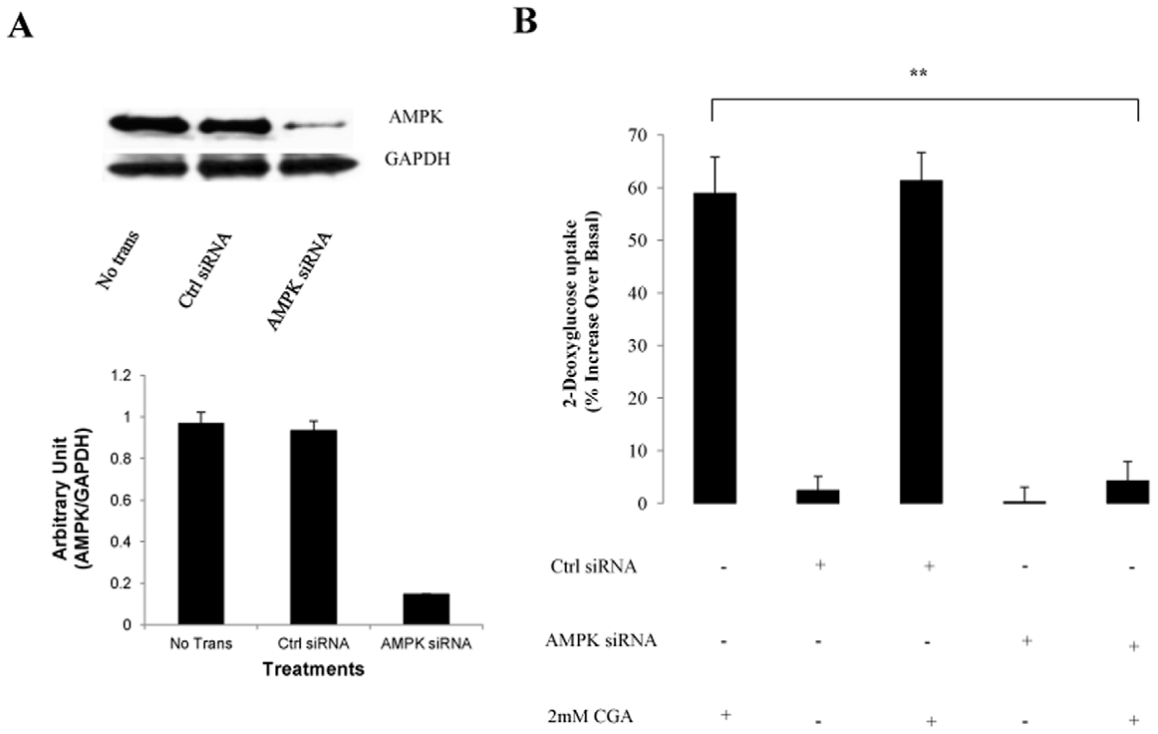
Effects of gene silencing of AMPK on CGA-stimulated glucose transport in L6 myotubes. L6 myotubes were transfected with vehicle, unrelated siRNA or AMPKα1/2 siRNA as described in Research Design and Methods. A: Expression of AMPKα1/2 after transfection with or without unrelated siRNA or AMPKα1/2 siRNA. B: Transfected or non-transfected myotubes were incubated with 2 mmol/l CGA for 24 hours. 2-deoxyglucose uptake was measure over a 30-minute period using liquid scintillation method. Readings were expressed as percentage increase over basal uptake that was obtained from non-transfeted cells incubated with vehicle. Results are the mean ± SE of three independent experiments. **P<0.01 compared with non-transfected-control treated with CGA.

Besides using inhibitor compound c, AMPKα content was reduced with RNA silencing. AMPKα 1/2 siRNA nucleotides reduced the total expression of AMPKα 1/2 by 74±7% compared with transfection with equal concentration of unrelated control siRNA sequence (Fig 4B). Transfection with AMPKα 1/2 or unrelated siRNAs alone did not affect glucose transport. However, reduction in total expression of AMPKα 1/2 significantly reduced glucose transport stimulated by CGA by 58±5% (Fig. 4A). Unrelated control siRNA did not significantly affect CGA-stimulated glucose transport, though a slight increase was detected. Taken together, the data suggested that AMPK may have a major contribution to CGA-induced stimulation of glucose transport.

### CGA increased GLUT 4 translocation, most possibly through AMPK activation

**Figure 5:**
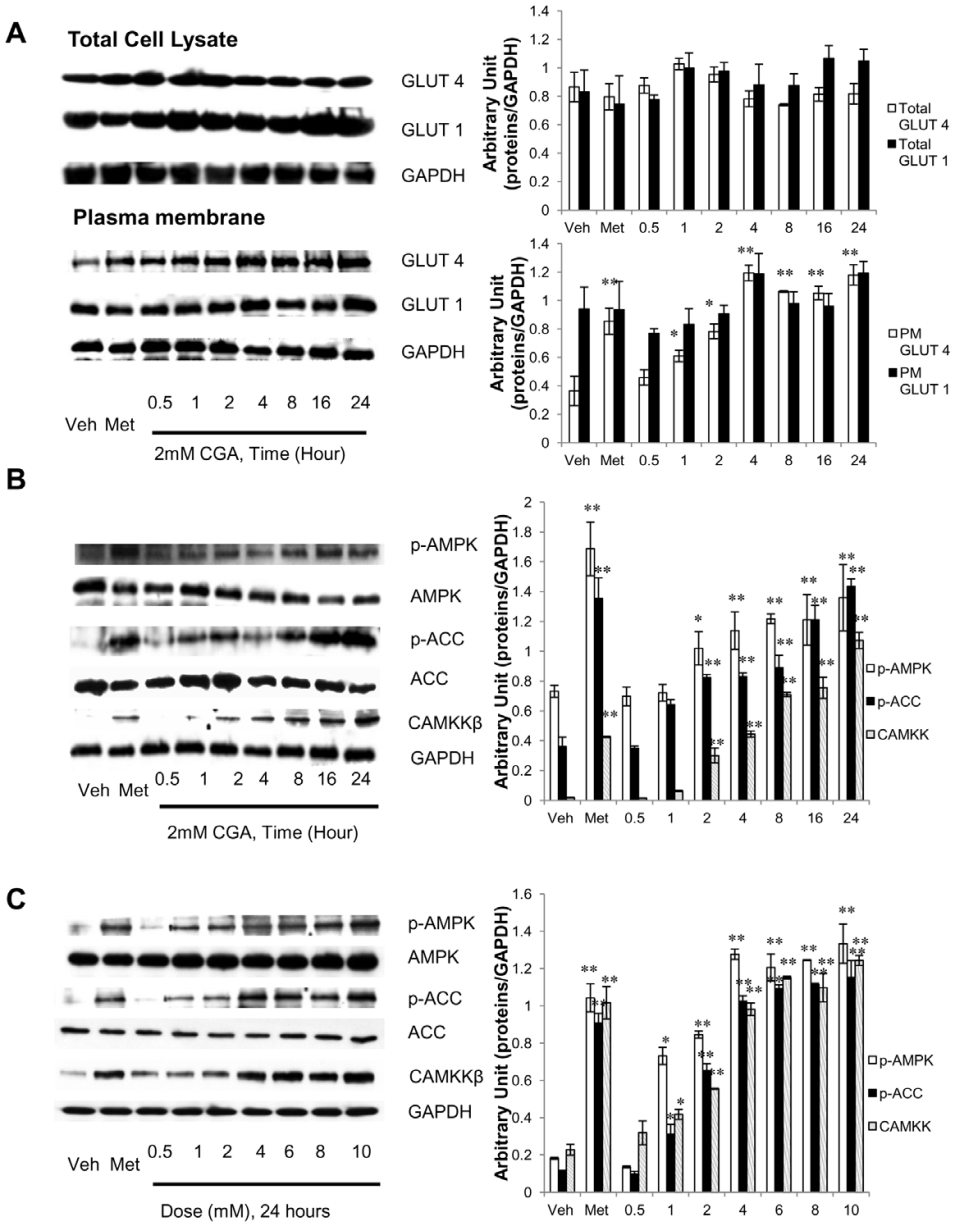
Dose- and time- dependent phosphorylation of AMPK. A: Myotubes were treated with 2 mmol/l CGA for various incubation periods up to 24 hours. Plasma membranes were isolated and detected for GLUT 4 and GLUT 1 through immunoblotting. B: Myotubes were treated with 2 mmol/l CGA for various incubation periods up to 24 hours. C: Myotubes were treated with incremental doses of CGA for 24 hours. Whole cell lysate was used for the detection of p-AMPK, AMPK, p-ACC, ACC and CaMKKβ. Illustrated are the representative images of three independent experiments. Results are the mean ± SE of three independent experiments. *P<0.05, **P<0.01 compared with controls.

**Figure 6:**
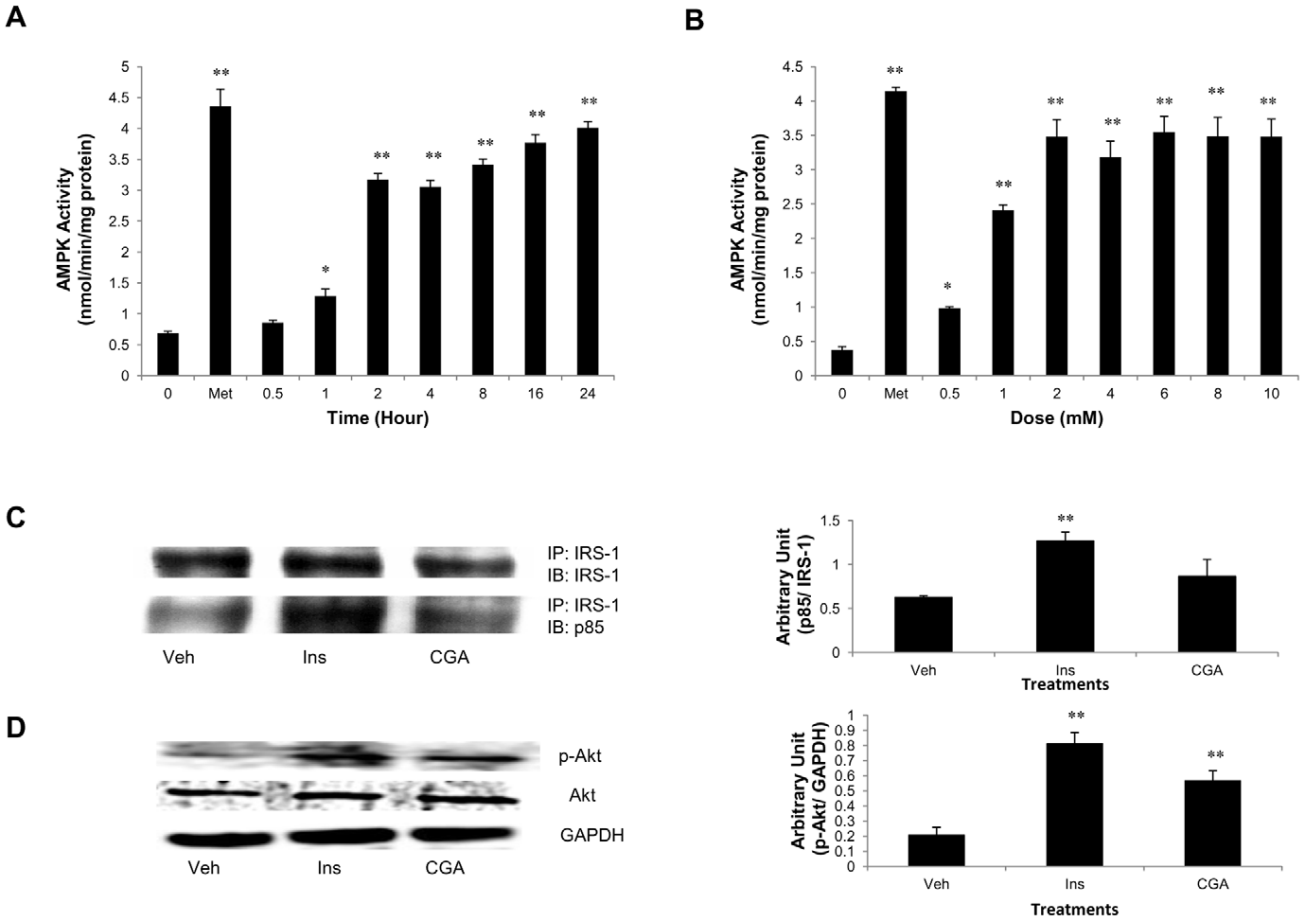
Increased AMPK activity and Akt phosphorylation in the absence of PI3K activation by CGA. A: Myotubes were treated with 2 mmol/l CGA for various incubation periods up to 24 hours. B: Myotubes were treated with 2 mmol/l CGA for various incubation periods up to 24 hours. Whole cell lysate was immunoprecipitated with anti-AMPK α1/2. Immunoprecipitate was assayed against SAMS peptide in the presence of [γ-^32^P]ATP. Kinase activity was expressed as incorporated ATP/mg protein/minute. C: Myotubes were treated with vehicle, 100 nmol/l insulin or 2 mmol/l CGA. Whole cell lysate was immunoprecipitated with IRS-1 and immunoblotted for IRS-1 and p85 subunit of PI3K. D: Myotubes were treated with vehicle, 100 nmol/l insulin or 2 mmol/l CGA. Whole cell lysate was detected for p-Akt through immunoblotting. Results are the mean ± SE of three independent experiments. *P<0.05, **P<0.01 compared with controls.

Glucose transport is mediated by the members of GLUT protein family, which consists of 12 transmembrane transporters [30]. We therefore investigated whether the glucose transport in response to CGA was accompanied by an increase in translocation of the two major transporters responsible for tissue glucose disposal, GLUT 1 and/or GLUT 4, to the plasma membrane of myotubes. Treatment of myotubes with CGA for different time periods augmented GLUT 4 content on plasma membrane of myotubes, with the significant increase first observed at 1-hour incubation, increasing in a time-dependent manner for up to 24 hours (Fig. 5A). Metformin demonstrated a similar increase in GLUT 4 on plasma membrane of myotubes. However, neither metformin nor CGA caused significant changes in total GLUT 4 expression or plasma membrane-GLUT 1. AMPK activation has been associated with increased GLUT 4 translocation to plasma membrane and enhanced glucose transport [31], [32]. We have shown that AMPK is vital for CGA to enhance glucose transport. Thus, to further show that CGA activates AMPK, we evaluated the effect of CGA on AMPK phosphorylation and likewise its downstream substrate, ACC. Like metformin, CGA increased AMPK phosphorylation on T172 and also ACC phosphorylation on S79 in a time- (Figure 5B) and dose-dependent (Figure 5C) manners. In addition, AMPK activity was increased time- and dose-dependently also by CGA and therefore explaining its phosphorylation stimulated by CGA (Figure 6A). Several upstream signaling were known to phosphorylate AMPK such as cellular stress that causes a fall in ATP: AMP ratio [33], tumor suppressor kinase LKB-1 [34] and CaMKK [35]. We here for the first time showed that activation of AMPK by CGA was mediated by CaMKK (Figure 5B & 5C).

### CGA did not induce association of p85 subunit of PI3K to IRS-1

Activation of PI3K requires association of its p85 subunit to IRS-1 as shown by insulin in Fig. 6C. However, CGA did not produce any significant effect on the association of p85 to IRS-1 immunoprecipitates of myotubes compared to the association observed in vehicle-treated myotubes. This again suggests that the stimulatory effect of CGA on glucose transport may not be attributable to PI3K activation. To further examine whether CGA activates the PI3K-Akt pathway, we investigated the effect of CGA on Akt phorphorylation. Surprisingly, we observed that Akt was phosphorylated at Thr 308 by insulin as well as CGA (Fig. 6D).

### Effect of CGA on cell viability and cell proliferation

**Figure 7:**
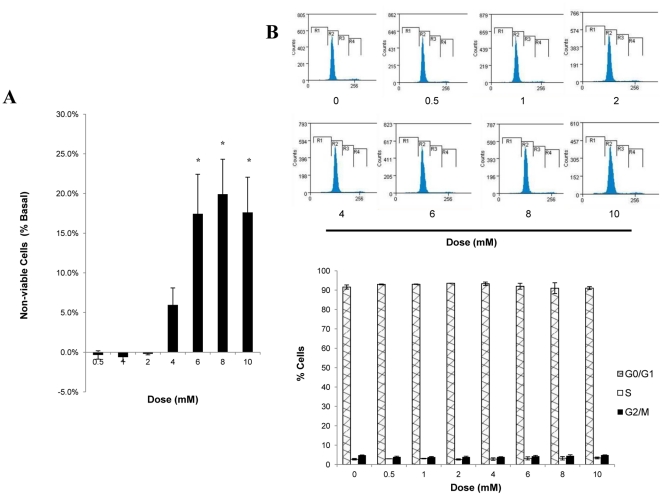
Effect of CGA on cell viability and cell proliferation of L6 myotubes. Myotubes were incubated with incremental concentrations of CGA for 24 hours. A: Viability of myotubes was measured using MTT staining. Readings are expressed as a percentage of non-viable cells compared to vehicle-treated myotubes. B: Numbers of cells in cell-cycle phases were examined using propidium iodide staining and FACS analysis. Readings are expressed as percentage of cells stained by propidium iodide at different phases. Results are the mean ± SE of three independent experiments. *P<0.05 compared with vehicle-treated control.

To discard the possibility that increases in glucose transport in response to CGA might be due to the changes in myotube numbers, we assessed the effect of increasing doses of CGA on cell viability and cell proliferation. No significant changes were observed up to a concentration of 4 mmol/l (Fig. 7A). However, significant increases in cell death were observed at 6 mmol/l (17.4±5%), 8 mmol/l (19.9±4%) and 10 mmol/l (17.6±4%) compared to vehicle-treated myotubes. On the other hand, myotubes treated with different concentrations of CGA did not produce any significant changes in all three cell-cycle phases, G0/G1, S and G2/M, compared to the vehicle-treated myotubes (Fig. 7B). Taken together, at the concentration of 2 mmol/l, CGA did not cause significant cell death or cell-cycle arrest, thus eliminating the possibility that cell numbers may affect the study of glucose transport.

## Discussion

The estimated prevalence of diabetes among adults in 2001 was 171 million people and was expected to increase to 366 million people by 2030 [36]. Majority of the diabetic cases, especially of Type 2 diabetes, are associated with diet and lifestyle managements. Coffee holds second position in consumption among all non-alcoholic beverages after water, and people from all over the world consume approximately 500 million cups of coffee every year [37]. An inverse relation between coffee and type 2 diabetes has been reported [1], [2], [3], [4], [5], [6] and it could not be explicated by caffeine, magnesium, and blood pressure [38]. Previous studies of CGA, the second abundant component in coffee after caffeine, showed its ability to delay intestinal glucose absorption and inhibit hepatic glucose output [15], [16], [17], [21]. There is no study to implicate CGA to peripheral glucose disposal until Prabhakar and Doble showed for the first time that CGA stimulated glucose transport in myotubes via increased expression of GLUT 4 and PPAR-γ transcript [22]. However, merely increase in GLUT 4 gene expression cannot explain the increase in glucose transport and the study lacks clarification on the mechanisms involved to enhance glucose transport in skeletal muscle.

In the present study, we showed for the first time the effect of CGA on fasting blood glucose in a diabetic animal model. db/db mice with homozygous spontaneous mutation of leptin receptor is a diabetic model showing Type 2 diabetic characteristics such as obese, uncontrolled rise in blood glucose and insulin. Intraperitoneal injection of CGA lowered the fasting blood glucose in db/db mice and this is consistent with the findings from Van Dijk et al. (2009) in overweight patients [14]. In addition, we found out that this effect continued to last for another 30 minutes after the diabetic animals were challenged with 2 g/kg of glucose. We believed that although its duration of action is relatively short compared to metformin, long-term consumption of CGA may be beneficial as seen in coffee consumption. Further long-term study is required to support this hypothesis. Decrease in fasting blood glucose can be due to increased glucose disposal in peripheral tissues such as skeletal muscle. Hence, soleus muscle was isolated from db/db mice and treated with CGA followed by 2DG transport assay. CGA was shown to stimulate and enhance both basal and insulin-mediated glucose transports and thus augmenting glucose utilization in the muscle. Addictive effect of CGA in insulin-mediated glucose transport suggests that CGA may act through a significant pathway which is different from insulin signaling.

To further support the data we found using the db/db mice, we investigated the effect of CGA on glucose transport in L6 myotubes and the possible mechanisms to execute its function in glucose transport. The results of our study showed that CGA stimulated glucose transport in L6 myotubes in a dose- and time-dependent manner. Optimum increase (∼1.5 fold) was observed at the dose of 2 mmol/l after 24-hour incubation period, compared to vehicle-treated control. Using non-radioactive glucose transport assay, Prakhabar and Doble (2009) demonstrated that CGA caused significant increase of glucose transport at micromolar concentration. However, we found that micromolar amounts of CGA only mildly stimulated glucose transport (data not shown). To examine the mechanism by which CGA exerts its effect on glucose transport, we used several selective glucose transport inhibitors, such as wortmannin and compound c, which inhibit PI3K and AMPK respectively. We found that wortmannin did not affect CGA-stimulated glucose transport whereas compound c significantly decreased glucose uptake stimulated by CGA. In addition, AMPK knockdown also abolished the stimulatory effect caused by CGA on glucose transport of myotubes. Taken together, these results suggest that the effect of CGA on glucose transport may possibly be mediated by AMPK.

AMPK is a master sensor and regulator of cellular energy balance [39]. It is activated by various pharmacological, pathological and metabolic stressors such as metformin, thiazolidinediones, hypoxia and exercise. Activation of AMPK leads to translocation of GLUT 4 from intracellular membranes to plasma membranes, thus increasing glucose transport [40]. Consistent with this, our results showed a time- and dose-dependent increase in phosphorylation of AMPK and likewise its activity, leading to an increase in the number of GLUT 4 on the plasma membrane of myotubes. However, no changes were observed on the translocation of GLUT 1, suggesting that CGA may only affect insulin-sensitive tissues such as skeletal muscles, liver and adipocytes. Calmodulin-dependent protein kinase kinase-β (CaMKKβ) has been reported as an alternative upstream kinase for AMPK besides cellular stress and LKB-1 [35]. We examined whether CGA causes any changes in ATP: AMP ratio but no significant changes were observed (data not shown). We found out later that CGA increased expression of CaMKKβ and this may be the upstream target of CGA that activates AMPK. In accord with the earlier report [22] that CGA did not show any significant effect on the expression of PI3K, we further demonstrated that CGA did not activate PI3K as no significant association was observed between p85 subunit of PI3K and IRS-1, suggesting a negative role of CGA on phosphorylation of IRS. However, to our surprise, we observed phosphorylation of Akt in the absence of PI3K activation. In rat heart, phosphorylation of Akt has been shown to phosphorylate AMPKα1 subunit at Ser 485/491 a hence interfering with the LKB-1-mediated activation of AMPK at its Thr 172 residue [41]. This indicates that Akt negatively influenced AMPK activation and activity. Other groups of researchers have reported that several stimuli such as adiponectin [42] and VEGF, [43] elicited parallel activation of both AMPK and Akt. Likewise, they proposed the upstream nature of AMPK, by which Akt is activated by the phosphorylation of AMPK at Thr 172. This is highly notable as both insulin-dependent and AMPK-dependent pathways converge to activate Akt substrate of 160 kDa (AS160) and Akt may be the point of convergence instead of AS160. Our results seem to favour the latter but subsequent studies to determine the activities of AMPK and Akt are required to support the occurrence of this phenomenon.

Several studies have reported that AMPK activation by the well-known AMPK activator, AICAR, led to cell cycle arrest which involved accumulation of tumor suppressor protein p53 [44], [45]. However, we did not detect any significant changes on cell viability and cell cycle of L6 myotubes even after 24 hours of incubation in the presence of 2 mmol/l CGA. This might be due to the parallel activation of pro-survival Akt, as Akt regulates apoptosis through direct targets such as Bad, caspase 9, the Forkhead family of transcription factors and the NF-κB regulator, IKK [46]. More importantly, the data eliminates the possibility that the increase in glucose transport might be due to the effect of CGA on cell proliferation.

In summary, we have demonstrated that CGA decreases fasting blood glucose in db/db mice using a glucose tolerance test. Also, CGA stimulated glucose transport in skeletal muscle through the translocation of GLUT 4, mediated by the activation of AMPK. At 2 mmol/l, CGA did not cause any deleterious effect on cell viability and cell proliferation. As a second major component in coffee, these data, together with those shown by other studies [15], [16], [17], [20], [21], [22], strongly suggest that CGA may contribute, at least in part, to the beneficial effect of coffee in blood glucose level of patient with Type 2 diabetes. However, further experiments are needed to support our hypothesis that CGA increases peripheral glucose disposal in skeletal muscle and therefore lowering fasting blood glucose.
